# Artificial Neural Network-Based Prediction of the Optical Properties of Spherical Core–Shell Plasmonic Metastructures

**DOI:** 10.3390/nano11030633

**Published:** 2021-03-04

**Authors:** Ehsan Vahidzadeh, Karthik Shankar

**Affiliations:** Department of Electrical and Computer Engineering, University of Alberta, 9211-116 St, Edmonton, AB T6G 1H9, Canada; vahidzad@ualberta.ca

**Keywords:** core–shell nanoparticles, metal-semiconductor heterojunctions, plasmonic catalysis, metal oxides, artificial intelligence

## Abstract

The substitution of time- and labor-intensive empirical research as well as slow finite difference time domain (FDTD) simulations with revolutionary techniques such as artificial neural network (ANN)-based predictive modeling is the next trend in the field of nanophotonics. In this work, we demonstrated that neural networks with proper architectures can rapidly predict the far-field optical response of core–shell plasmonic metastructures. The results obtained with artificial neural networks are comparable with FDTD simulations in accuracy but the speed of obtaining them is between 100–1000 times faster than FDTD simulations. Further, we have proven that ANNs does not have problems associated with FDTD simulations such as dependency of the speed of convergence on the size of the structure. The other trend in photonics is the inverse design problem, where the far-field optical response of a spherical core–shell metastructure can be linked to the design parameters such as type of the material(s), core radius, and shell thickness using a neural network. The findings of this paper provide evidence that machine learning (ML) techniques such as artificial neural networks can potentially replace time-consuming finite domain methods in the future.

## 1. Introduction

There is tremendous research interest in the investigation of plasmonic phenomena in and around coinage metal nanoparticles [1]. Plasmonic nanoparticles possess interesting optoelectronic properties such as tunable optical resonances, production of hot carriers through plasmon decay, local electromagnetic field enhancement and the Purcell effect, which make them useful in a gamut of applications including surface-enhanced Raman scattering (SERS), light emission, imaging, sensing, photovoltaics and photocatalysis [2,3,4,5]. The optical properties of nanoparticles made of coinage metals (Au, Ag and Cu) are known to be sensitive to the particle size, particle shape and the environment surrounding the particle. A variety of plasmonic metals with different shapes such as spheres, cubes, stars, octahedra and triangles with different optical properties have been synthesized [6,7]. Amongst all these shapes, spheres are the easiest to fabricate while simultaneously achieving a monodisperse size distribution and preventing aggregation. 

The usage of plasmonic coinage metal nanoparticles in catalysis and sensing is dramatically hindered by their physico-chemical properties, such as the chemical and photochemical reactivity in electrolytes and oxidizing environments, the poor adsorption of reactant atoms over noble metal surfaces, the inability to resist high temperature without melting, reaction or shape change, poor separation and utilization of hot carriers generated by plasmon decay, etc [8]. The chemical/photochemical stability, abrasion resistance and thermal resistance of coinage metals can be improved by applying a protective layer (shell) of ceramics such as metal oxides and nitrides [2,8,9,10]. When the said ceramic protecting the plasmonic core from the environment also has semiconducting properties, a metal-semiconductor heterojunction is created. The built-in electric field associated with such a metal-semiconductor heterojunction is a highly effective method to separate oppositely charged carriers and enhance the charge transfer efficiency of a plasmonic hybrid nanoparticle [11,12]. Semiconductor shells typically have a high frequency dielectric constant higher than 5, which is complementary to the negative relative permittivity exhibited by the coinage metals over the visible and near-infrared spectra range. Metal oxides also show strong chemisorption of a number of reactant molecules including water, CO_2_, CO and a host of organic molecules important in industrial heterogeneous catalysis. Thus, the fabrication of such core–shell plasmonic meta-atoms results in a hybrid that inherits the useful properties of both of the parent materials with unique optical, chemical and mechanical properties which the individual parent materials would not possess alone [13,14]. All these advantages enable core–shell plasmonic nanoparticles to offer tunable, high Q-factor optical resonances, efficient charge transfer, efficient harvesting and utilization of hot carriers, strong interaction with reactant molecules, and good chemical and physical stability in different environments for different applications [3,15,16]. Consequently, the investigation of the optical properties of the core–shell hybrids is of great importance [15,16,17].

Since a number of degrees of freedom (choice of the core and shell materials, core radius and shell thickness) exist in the fabrication of the core–shell hybrid, the problem of inverse design of a perfect core–shell structure with a desired far-field optical response has recently attracted more attention [18,19]. The inverse design can map the required far-field optical response of a core–shell structure with the material properties. The inverse design approach accelerates the design process and obviates the need for repetitive simulations to reach the final far-field response [14,16,20].

**Figure 1:**
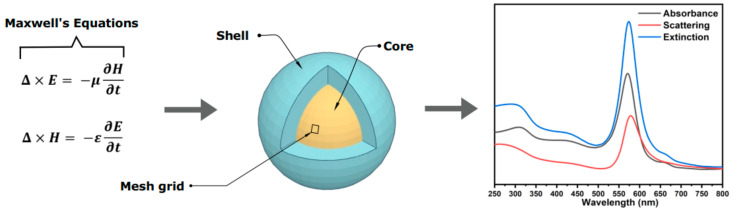
Schematic diagram representing the working principle of FDTD simulation.

Simulation approaches are utilized to investigate the optical properties of core–shell hybrid systems and optimize their optical properties before fabrication to ensure that resources such as researcher time, energy, fabrication, and characterization costs, etc., are used minimally and efficiently. The finite difference time domain (FDTD) method is the most convenient way to simulate the light–matter interactions [21]. In the FDTD method, the shape of the nanoparticle and its constituent materials are defined first, then the FDTD engine uses meshing grids to solve Maxwell’s equations on tiny blocks of the simulation environment (Figure 1). The far-field response of a simulation target including absorption, scattering and extinction spectra are among the most important pieces of information, which can be obtained through FDTD simulations [1,21].

Even though FDTD simulations constitute a powerful tool to obtain the near-field and far-field optical response of the nanoparticles, this tool has its own shortcomings as well. Since FDTD is a finite difference method, the computational cost of this method is extremely dependent on the mesh size and the size of the simulation region. The following equations describe the relation between the computational expense and the simulation time with the mesh size and over all size of the simulation region [22]:

(1) Memory requirements∼V1dx3

(2) Simulation Time∼V1dx4

Where dx is the mesh step size and V is the volume of the simulation region. These two equations shed light on the extreme dependence of the finite difference methods on the mesh size, a dependence that makes the FDTD method a time consuming and computationally expensive method, which requires both time and enough memory resources to converge [20,23,24]. As a result, FDTD methods are considered slow by today’s standards especially for simulation of light–matter interactions for the huge nanostructures [24].

The 21st century is likely to be the century of automation. Using prior experience from the industrialization era, humankind already knows that automating a process is the key to reducing its cost and improving its efficiency. From the advent of computing machines in the mid 19th century, computers began to have an increasing impact on civilization and the everyday lives of humans. Artificial intelligence and machine learning represent the next frontier of computers able to think and learn, whose application in the present time extends from autonomous driving to the virtual assistant in our smartphones. Recently, artificial neural network (ANN)-based machine learning algorithms were successfully utilized in applications such as computer vision, speech recognition, autonomous driving, etc [20,23]. ANN is reported to successfully predict the outputs of the computationally expensive finite difference methods to solve partial differential equations (PDEs) [25,26] in applications ranging from heat transfer [27] to stress prediction [28] with little to no computational expense [29]. Nelson and Di Vece trained a neural network using FDTD simulation results to help optimize the optical absorption of halide perovskite solar cells containing core–shell Ag nanoparticles [30]. Bravo-Abad and colleagues reviewed the use of deep learning approaches in nanophotonics to perform the nonlinear mapping of material geometry and composition with the resulting functional properties [18]. Inspired by these ideas, herein we successfully trained different ANNs with different architectures for ultrafast prediction of the far-field optical responses (including absorbance, scattering and extinction) of plasmonic core–shell materials in the first step. In the second step, we tried to address the inverse design problem using a unique ANN architecture, which can predict the material characteristics needed to obtain a desired far-field response.

## 2. Methods

### 2.1. FDTD Simulations

The Lumerical^®^ (Ansys, version: 8.24.2466) software package was used to obtain the input data for the ANNs. The user-friendly environment provided by the graphical user interface (GUI) of Lumerical software enables one to easily simulate light–matter interactions. In addition, Lumerical’s GUI supports scripting commands, which facilitate the automatic simulation of a batch of predefined simulations. The built-in material library of Lumerical contains the optical constants of the most important semiconductors, metals and dielectrics used in optoelectronic devices. All of these have made Lumerical one of the most widely used programs for electromagnetic simulation applications. The simulations in this work were performed using various combinations of the core and shell materials with different thicknesses. For the core–shell structures, Au, Ag and Cu (3 different core materials), which are the most important plasmonic metals [31] were selected as the materials constituting the core while TiO_2_ [32], ZnO [33], InAs, InP and GaAs (5 different shell materials), which are the most important semiconductor materials used in optoelectronic devices were chosen as the shell materials. The materials used in this study along with their indices are reported in Table 1. 

Since TiO_2_ and ZnO were not defined in the built-in materials database of Lumerical, they were added to the materials database using the wavelength-dependent refractive indices reported in references [32] and [33], respectively. The environment surrounding the core–shell structures was air (i.e., refractive index of the environment was set to 1). A total field scatter field (TFSF) source with a bandwidth of 250–800 nm was added to the simulation environment as a light source while absorption cross-section monitors were used to record the absorption and scattering spectra. A uniform mesh with a size of 5 nm were rendered by the GUI and a more accurate mesh override with maximum size of 2 nm on the surface of the core–shell structure was introduced later to increase the accuracy of the calculations. Since the core–shell geometry is symmetrical, a symmetrical boundary condition in the x direction and anti-symmetrical boundary condition in the y direction were imposed to increase the speed of the simulation. For the core–shell structure 10 different radii were chosen in the range 2.5–50 nm and 10 different shell thicknesses were chosen in the range 2.5–25 nm. This gave a total of 3 × 5 × 10 × 10 = 1500 different simulated spectra. To obtain a smooth spectrum, the results of the monitors were collected in 1000 data points, which were associated with the TFSF light source’s wavelength. The simulations in total yielded 1500 × 1000 = 1,500,000 data points for each of the absorption, scattering and extinction batch simulations. These simulated data were used as the feed for the ANNs convergence in the next step.

### 2.2. ANN Architectures

**Figure 2:**
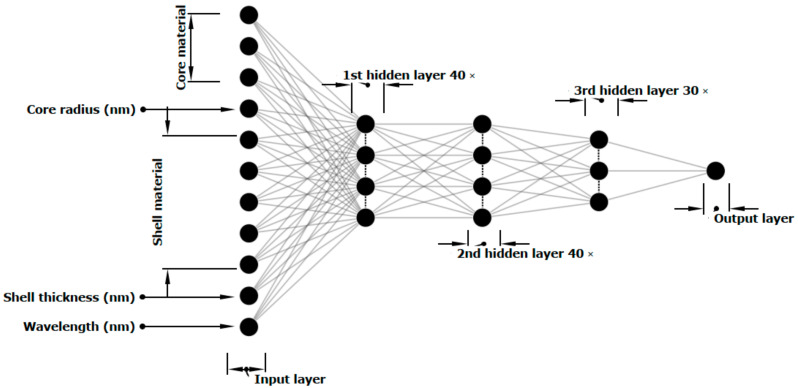
Schematic diagram representing the architecture of the APN used for predicting the absorption spectra of the plasmonic core–shell materials.

Three different ANNs with three different structures were designed to predict the far-field optical responses: the absorption prediction network (APN), the scattering prediction network (SPN) and the extinction prediction network (EPN). The results obtained by FDTD simulations were used as the input for training the three ANNs. The input data in all these ANNs were the combination of both categorical data (e.g., Au and ZnO as the materials used in the core and shell simulations) and quantitative data (e.g., the size of the core radius or the shell thickness), in order to make the input data interpretable for the ANNs. Hot encoding was used to convert the categorical data into binary (0 or 1) format. Since the simulations were performed using 3 different core materials and 5 different shell materials, the hot encoding results converted these two categorical values into 5 + 3 = 8 binary values. In addition to this, the radius of the core, the shell thickness, and the wavelength of the TFSF light source were also given as the input features to the neural network, which made it 11 different input features in total. Figure 2 exhibits the 3 hidden layer ANN architecture used for the absorption spectrum prediction (APN). The 11 features were fed to the neural network as inputs, the 3 hidden layers had 40, 40 and 30 neurons (3381 parameters in total) respectively and the output was a value between zero and 1 corresponding to the value of the absorption in that specific wavelength. The architecture of ANNs for scattering and extinction was the same with different neurons in each hidden layer, the three hidden layers in SPN had 80, 80 and 80 nodes (14,001 parameters in total) and the three hidden layers in EPN had 80, 80 and 120 neurons (17,281 parameters in total), respectively. 

Since the outputs of neural network should be compared to the actual values from the simulations, the problem is one of regression. The error functions for all the ANNs were set to the mean squared error (MSE, Equation (3)) where y_predict_ is the value predicted by the neural network and y_actual_ is the value simulated by the neural network. The tanh function was chosen as the activation function in all three layers of ANNs and the activation function of the last layer was a linear activation function. To facilitate the convergence, the input features were normalized to the values between 0 to 1 prior to training the ANNs. In addition, 20% of the data was set aside as the test set and the training of the ANNs was conducted on the 80% of the available data. The architecture of all the ANNs and their hyper-parameters, such as the number of hidden layers, number of neurons in each layer, batch size, the ratio between the train set and the test set and the active function were optimized for each of the ANNs, and the final architectures are reported above.

(3) $$\mathrm{MSE}=\;\frac{1}{n}\underset{n}{\sum }{\left(y_{\mathrm{predict}}-y_{\mathrm{actual}}\right)}^{2}$$

The inverse design problem is slightly more complicated than the far-field optical response prediction. While optical response prediction can simply be categorized as a regression problem, the inverse design should successfully predict both the categorical (i.e., type of the materials used as core and shell) and continuous numerical (i.e., core radius and shell thickness) values. The prediction of categorical values is a classification problem while prediction of the numerical values of the geometric parameters of the core–shell structure falls in the category of regression problems. To address this issue for the inverse design problem, we developed two different architectures—a multi-class classifier inverse design network (IDN-Classifier) and a regressor inverse design network (IDN-Regressor). The simulated absorption spectra of the plasmonic core–shell structures (1000 datapoints for each absorption spectrum) were used as the far-field optical response to feed the IDN networks and the expected outputs of the IDNs were two datapoints associated with the core radius and shell thickness for IDN-Regressor and eight categorical outputs (material of choice for the core and shell) for IDN-Classifier.

**Figure 3:**
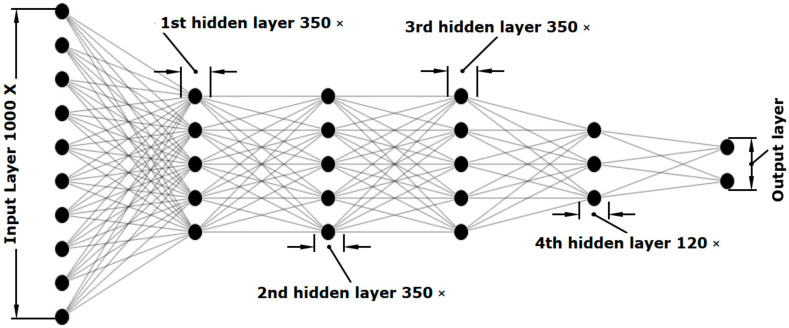
Schematic diagram representing the architecture of IDN-Regressor used for inverse design problem.

Figure 3 illustrates the architecture of the IDN-Regressor. The IDN-Regressor consists of 4 hidden layers with the first three layers containing an identical number of neurons (350, 350, 350 and 120 neurons). The architecture of IDN-Classifier was the same with 250 neurons for the fourth hidden layer. The loss function for the IDN-Regressor was set to MSE (Equation (3)) and the loss function for the IDN-Classifier was set to binary cross entropy (BCE, Equation (4)), where $\sigma \left(y_{\mathrm{predict}}\right)$ is the sigmoid function. The number of trainable parameters were 639,138 and 685,808 for the IDN-Regressor and IDN-Classifier respectively. The tanh activation function was chosen as the active function of each layer, except for the last layers for both classifier and regressor inverse design networks. For the last layer, the activation function of the IDN-Regressor was set to the linear activation function and the activation function for the IDN-Classifier part was set to a sigmoid activation function. The optimizer was set to Adam optimizer with a learning rate of 0.001. All the hyperparameters, such as the number of hidden layers, the number of neurons in each layer, optimizer type and learning rate of the optimizer, were optimized to minimize the error with proper caution to avoid overfitting.

(4) $$\mathrm{BCE}=-\left[y_{\mathrm{actual}}\log\;\sigma \left(y_{\mathrm{predict}}\right)+\left(1-y_{\mathrm{actual}}\right)\log\left(1-\sigma \left(y_{\mathrm{predict}}\right)\right)\right].$$

## 3. Results and Discussion

### 3.1. Predicted Absorption, Scattering and Extinction Spectra by ANNs on the Previously Seen and Unseen Data

**Figure 4:**
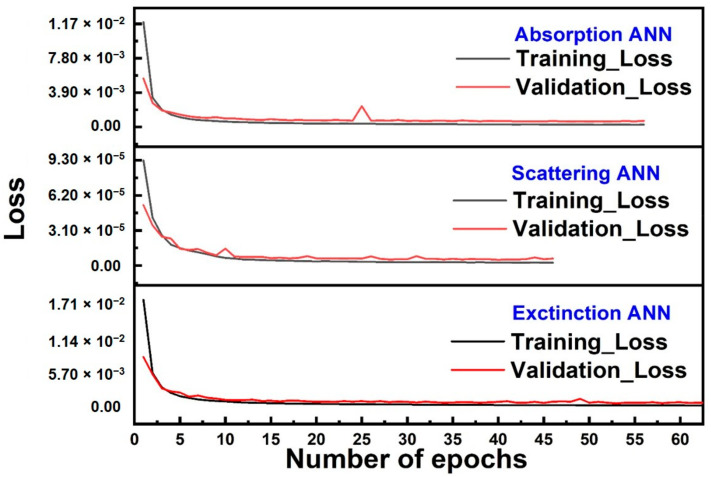
The loss functions of absorption, scattering and extinction as a function of the number of epochs.

The training process for each of the ANNs was performed using the optimized hyper-parameters and the maximum number of epochs was set to 500. A call back function, which set aside 10% of the training data with a patience of 10 for the validation loss, was used to control the number of epochs for the training process of each of the ANNs. Figure 4 exhibits the training and validation loss as a function of number of epochs for APN, SPN and EPN. The training loss function exhibited a gradual decrease as the number of epochs increases and reached stable values in the range of 10^−4^ for the absorption and extinction ANNs and in the range of 10^−6^ for the scattering ANN beyond 10 epochs. The validation loss function also exhibited the same trend as the number of epochs increased and reached stable values in the range of 10^−4^, 10^−4^ and 10^−6^ for the APN, EPN and SPN, respectively. The fact that the value of validation loss is in close proximity of the training losses for all of the trained ANNs shows that overfitting did not occur for any of these trained ANNs. This was one of the ways to optimize the architecture of the ANNs because when the number of the hidden layers was increased beyond 3 layers, the same trend could not be seen for the validation loss function anymore. The optimized number of epochs determined by the call back function for the training process of the APN was determined to be 56 while for the SPN, the number of optimized epochs was 46 and for the EPN, the number of optimized epochs was 63. After the training was complete, the error of prediction for the test set was calculated to be 3.69 × 10^−4^, 1.12 × 10^−6^ and 1.85 × 10^−4^ for APN, SPN and EPN, respectively. As it has been mentioned in the ANN architecture section, the arcitecture of each ANN was optimized to reach the least amount of error for the test set. Table 2 exhibits a few different arcitectures for APN and their corresponsing errors on the test set as an example. These results show that a model with less than three layers cannot do a good job on predition on the test set. Also, when we increase the complexity of the APN from its ideal structure (increasing the number of layers or increasing the number of neurons in each layer), model tends to overfit and the test set error will increase.

**Figure 5:**
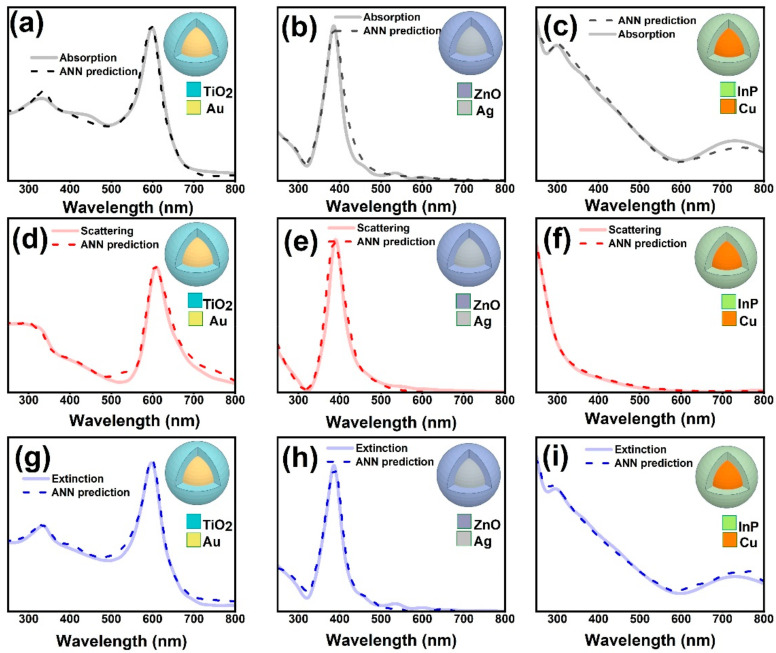
The FDTD-simulated and ANN-predicted absorption, scattering and extinction spectra of Au@TiO2 (**a**,**d**,**g**), Ag@ZnO (**b**,**e**,**h**), and Cu@InP (**c**,**f**,**i**) core–shell structures.

After the training was completed, the trained ANNs were used to calculate the absorption, scattering and the extinction spectra of the core–shell architectures. Figure 5 shows the comparison between the simulated, and ANN predicted, absorption, scattering and extinction spectra of a few randomly selected structures (with core radius and shell thicknesses already seen by the network). The inset of Figure 5 shows the materials chosen as the core and shell. In light of the fact that, on average, 20% of these data has never been used in the training or validation data sets, these results show an extremely acceptable agreement between FDTD simulation target spectra and the ANN predicted spectra. 

**Figure 6:**
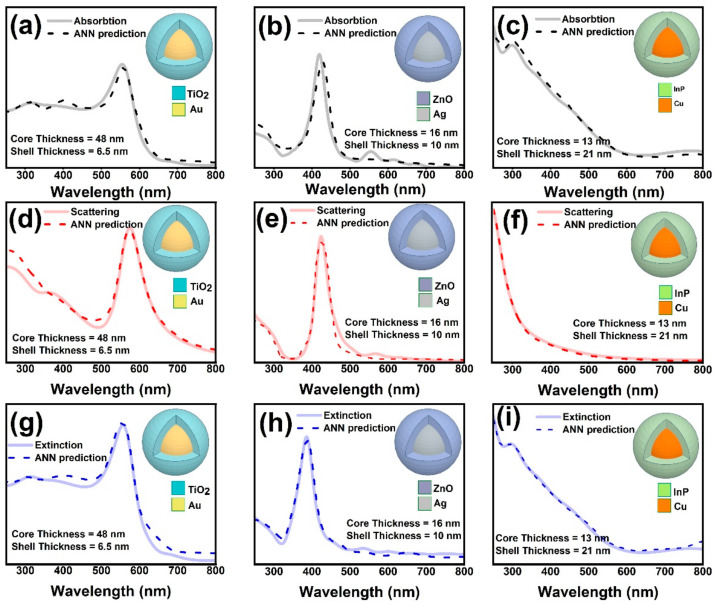
The FDTD simulated and ANN predicted absorption, scattering and extinction spectra of Au@TiO2 (**a**,**d**,**g**), Ag@ZnO (**b**,**e**,**h**), and Cu@InP (**c**,**f**,**i**) core–shell structures.

In order to examine the robustness of the trained ANNs in predicting the far-field optical responses of the core–shell structures, a few core–shell structures with features (core radii and shell thicknesses) that the trained ANNs did not get trained on at all were chosen to test the robustness of the trained ANNs. Figure 6 shows the predicted optical response of the robustness tests. The materials chosen for the core and shell and the corresponding radii and the thicknesses are reported in the inset of each figure constituting the panel in Figure 6. Interestingly, the results indicate excellent agreement between the FDTD simulated spectra and their ANN predicted counterparts. In the first instance, an Au@TiO_2_ core–shell metastructure with 48 nm core radius and 6.5 nm shell thickness was chosen. In this case, both the core radius and shell thickness are different from the core and shell thicknesses the ANNs were trained on. In the second instance, an Ag@ZnO core–shell structure was chosen wherein the shell thickness (10 nm) was chosen among the 10 shell thickness values the ANNs were trained on but the core radius (16 nm) was not among the core radii values used for training the ANNs. And in the last instance, a Cu@InP core–shell structure was chosen; in this instance the core radius (13 nm) was chosen among the 10 core radii the ANNs were trained on but the shell thickness (21 nm) was not among those thickness values used for training the ANNs. The fact that in all of these instances the trained ANNs could successfully predict the absorption, scattering and extinction spectra of the coreshell metastructures evidences the robustness of the trained ANNs and proves the fact that the training process yield a network that can actually predict each spectrum while not merely remembering or interpolating the previously seen data. 

### 3.2. The Inverse Design ANN

**Figure 7:**
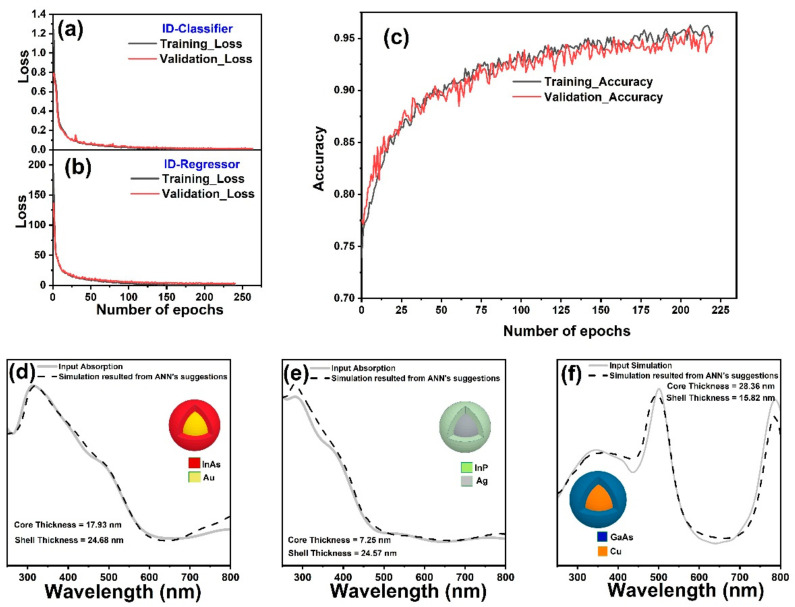
The training and validation losses of (**a**) IDN-Classifier network and (**b**) IDN-Regressor as a function of epochs (**c**) the training and validation accuracy of the IDN-Classifier as a function of number of epochs. (**d**,**e**,**f**) absorption spectra of Au@InAs, Ag@InP and Cu@GaAs core–shell structures which were randomly selected from test set, and corresponding simulated absorption spectra of the same core–shell structures with radius and shell thickness suggested by the IDN.

The training process for the IDN networks involved using the architecture described in previous section. The maximum number of epochs for both IDN-Regressor and IDN-Classifier were set to 500. The number of epochs were controlled using a callback function, which split 10% of the data to monitor the validation loss with a patience of 10. Figure 7a,b indicate the loss of IDNs as a function of number of epochs. Both the training loss and validation losses of the classifier and regressor networks decreased as the number of epochs increased. After 220 epochs, the classifier network’s loss reached the values of 0.003 and 0.0045 for the training and validation data sets while these numbers reached values of 0.61 and 0.78 the regressor network after 240 epochs. The fact that both training loss and validation loss followed the same trend shows that overfitting on training data did not occur for any of the IDN networks. After the training process finished, the errors for the prediction of test data set were calculated to be 0.005 and 0.73 for the IDN-Classifier and IDN-Regressor, respectively. 

For the IDN-Classifier, only monitoring the loss as a function of number of epochs is not enough to judge its performance. The accuracy of classification is an important decision factor for evaluation of the performance of a classifier network. Figure 7c exhibits the accuracy of classification for IDN-Classifier as a function of number of epochs for the training and validation data sets. The accuracy plot shows a gradual increase as the number of epochs increase and reached an excellent value of 95% and 94% for the training and validation data sets after 220 epochs. Keeping in mind that the goal of IDN was to predict the design parameters of the core–shell structure, which can mimic a desired absorption spectrum, three randomly selected spectra from the test set were chosen to find out if the design parameters suggested by the IDN network actually resulted in an absorption spectrum similar to the input absorption spectrum. The design parameters suggested by the IDNs for these three selected spectra were fed to Lumerical software to simulate the absorption spectrum. Figure 7d–f shows these three randomly chosen spectra and the FDTD simulated absorption spectra (dashed lines) using the IDN suggested design parameters. The inset(s) of the images in Figure 7 show the predicted core radii and shell thicknesses and materials suggested by the IDN. These results demonstrate the excellent performance of the IDNs in suggesting the design parameters for a core–shell structure with an optical response resembling the desired absorption spectrum.

### 3.3. Comparison between the Sspeed of FDTD Simulation vs Speed of ANN Prediction

**Figure 8:**
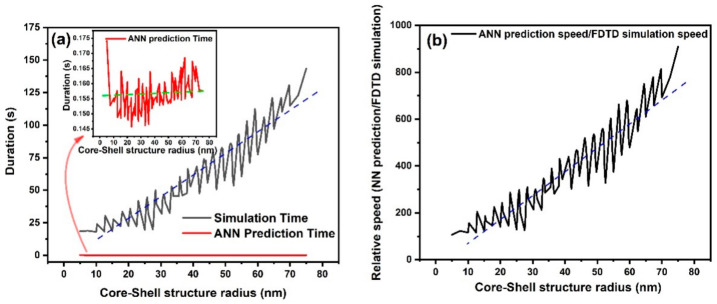
The FDTD simulation and ANN prediction time as a function of the radius of the core–shell structure (**a**) and the relative speed of the FDTD simulation and ANN prediction as a function of the radius of the core–shell structure (**b**).

During the simulation process, the simulation duration was recorded for each of the 1500 simulations. Based on Equations (1) and (2) the simulation time using FDTD method increases linearly with the size of the simulation region. For each specific core and shell material, there were 10 × 10 = 100 simulations (10 different radii and 10 different thicknesses). Au and TiO_2_ were chosen as the instances for the core and shell materials respectively and the duration of the 100 simulations using FDTD method as a function of the size of the combined core–shell structure (radius of the core + shell thickness) was monitored. Since the simulated radii of the cores was in the 2.5–50 nm range and the shell thicknesses were in the 2.5–25 nm range, the y axis of the graph is in the range of 5–75 nm. Keeping the core and shell materials constant (Au and TiO_2_), the ANN’s prediction duration for the same features used in the simulation, has been recorded. Figure 8a shows the relation between the FDTD simulation and ANN prediction durations with the size of the core–shell nanosphere’s radius. As shown in Figure 8a, the duration of the simulation with FDTD falls within the second domain while ANN prediction is in the millisecond range. The inset of Figure 8a exhibits the relation between the ANN prediction durations with the size of the core–shell structure’s radius in milliseconds domain. Furthermore, as mentioned in the introduction section, the FDTD simulation duration is dependent on the size of the simulation region (dashed blue line in Figure 8a is for guiding the eyes), which increases almost linearly with size of the core–shell structure. The inset of Figure 8a shows that a similar relation for the ANN prediction does not exist with the size of the structure since the prediction of the ANN is instantaneous and almost constant (green dashed line in the inset of the Figure 8a is for guiding the eyes.) This independence exhibits another advantage of the ANN prediction over the FDTD simulation, which is that not only is it faster than the FDTD simulation, its speed is also independent of the size of the structure. Figure 8b provides a comparison of the relative speed of the ANN predictions and the FDTD simulations and shows that for small structures, the speed of the ANN predictions can be 100 times faster than the FDTD simulations while for larger structures (75 nm) it can be almost 1000 times faster. Keeping in mind that the maximum size of the core–shell nanostructures was 75 nm in our study, these results show that for larger structures, the ANNs predictions can significantly outpace the FDTD simulation.

### 3.4. Significance for Nanomaterials Synthesis and Optimization, and Use in Functional Devices

The ability to quickly obtain the morphological parameters of a plasmonic nanomaterial with a desired optical response from an artificial neural network is a powerful enabler of cutting edge experimental research. One could envisage a number of applications where such information is decisive in design of experiments. For instance, LSPR-based sensors frequently use a metal oxide shell around an Au or Ag nanoparticle to both enable facile functionalization/chemisorption of analyte(s) on to the metal oxide surface and to protect the Au/Ag core from wear and corrosion. The source LED wavelength is typically known for sensor deployment and the core–shell nanomaterial needs to have a plasmon resonance well-matched to the light source to enable maximum refractive index sensitivity—this is one practical example of the inverse design problem. When the analyte in question is a biomolecule with a fluorescent tag and the fluorescence is to be detected or imaged, it is desirable to match the emission maximum or absorption maximum of the fluorophore with the plasmon resonance to achieve maximum local field enhancement of the light–matter interaction to boost sensitivity—another example of the inverse design problem. In broad-band light harvesting applications such as photovoltaics and photocatalysts, plasmonic nanoparticles are used to generate hot carriers and/or to maximize light–matter interactions. Furthermore, a thin insulating shell around the Ag/Au nanoparticle is needed to minimize carrier recombination on the metal surface. The ANN-guided inverse design of a core–shell plasmonic nanoparticle can then be used to boost the light absorption in spectral ranges where the active layer does not absorb efficiently and to achieve the maximum local electromagnetic field enhancement while still minimizing recombination.

## 4. Conclusions

Researchers throughout the world are finding novel applications for machine learning and broadening its horizons everyday. Recently its horizons have been reaching into simulations of the properties of nanomaterials as well. Electromagnetic phenomena are described by the four Maxwell’s equations. Finite difference methods (including FDTD), which are important tools for simulation of the optical properties of metamaterials rely on solving partial differential equations in tiny building blocks (meshes) of the defined structures, which renders them time consuming by today’s standards. In this report, we have shown that artificial neural networks (ANNs) with the proper architecture and optimized hyperparameters can rapidly predict the results of FDTD simulations. Plasmonic core–shell materials with different core and shell materials, and various core radii and shell thicknesses, are important materials for solar energy harvesting, sensing and light emission applications. We have chosen plasmonic metamaterials with sizes within the 5–75 nm range as a case study and our results show that using artificial neural networks, the far field optical response of the plasmonic core–shell materials can be rapidly predicted with high accuracy with little to no computational expense. Depending on the size of the whole core–shell structure, the speed of ANN prediction was estimated to be 100 to 1000 times higher than the FDTD simulations. The results from our work can be used as a proof of concept for potential replacement of FDTD simulation with faster artificial neural network methods. Furthermore, we have demonstrated that the problem of inverse design of a core–shell plasmonic metastructure with a desired absorption response can be easily tackled by a proper ANN structure. In general, the results from our work can be considered as a proof of concept for the next generation of electromagnetic simulations of nanomaterials, where rapid ANN can potentially replace sluggish finite difference methods.

## Figures and Tables

**Table 1:** Indices of the materials used in FDTD simulations.

None	Au	Ag	Cu	TiO_2_	ZnO	InAs	InP	GaAs
0	1	2	3	4	5	6	7	8

**Table 2:** The test error for APN networks with different architectures.

APN Architectures	Test Error
[40,40,30]	3.69 × 10−4
[80,80,60]	6.23 × 10−4
[100,100]	8.56 × 10−4
[40,40,30,30]	7.09 × 10−4

## Data Availability

The data presented in this study are openly available in [FigShare] at [https://doi.org/10.6084/m9.figshare.14141732.v1].
